# Trephining for Epilepsy

**Published:** 1889-11-16

**Authors:** 


### TREPHINING FOR EPILEPSY.

Dr. Williamson, physician to the Latchford Hospital, and Dr. Robert Jones, hon. surgeon to the Liverpool Stanley Hospital, publish a very interesting case of "trephining for epilepsy, with subsequent resection of trephine opening" (*British Medical Journal*, October 26th, 1889). The origin of the fits dated from injury to the head received two years ago, but the patient has an epileptic sister. After admission into hospital there was a constant succession of epileptiform seizures, so trephining was determined upon. The operation was performed at the site of the scar of the injury. A three-quarter inch trephine was employed. The circle of bone removed had attached to its internal surface a triangular piece of bone diving into the brain at right angles to the cranium to a length of three-quarters of an inch. A probe with a bent point was then passed into the trephine space, and anteriorly the inner plate felt bare. Another trephine hole was made a quarter of an inch anterior to the first, and the intervening ridge of bone removed by saw and forceps. This procedure yielded a small piece of necrosed bone. The wound was treated during the operation with perchloride of mercury, and afterwards covered with antiseptic dressings. For some four days after the operation the patient lay in a semi-comatose state, but occasionally becoming violent. About the fifth day he slept quietly and continued to improve, notwithstanding a hernia cerebri, until his discharge nearly two months afterwards. From the date of departure from hospital, January 15th, until April 28th the patient had no fit, but there was a little discharge off and on from the trephine wound. On the date last mentioned, after a little excess in drinking, a fit recurred. Attacks now took place daily, and pus was evacuated from the trephine wound. On May 6th a semilunar incision was made across the anterior limb of the former site. Having dissected the flap, Mr. Jones widened the sinus, and removed some of the fibrous filling of the trephine wound. At the bottom of the sinus, projecting brainwards, was a small spicule of necrosed bone. The subsequent history was satisfactory. He left the hospital in July perfectly well, and up to October 17th last had remained so. It should be remarked that the first operation was performed under very inferior surroundings, a small room and candle light, with no opportunity of sufficiently cleaning the scalp.

